# Illness presenteeism among physicians and trainees: Study protocol of a scoping review

**DOI:** 10.1371/journal.pone.0297447

**Published:** 2024-02-29

**Authors:** Lorenzo Madrazo, Jade Choo-Foo, Marie-Cécile Domecq, Kori A. LaDonna, Susan Humphrey-Murto

**Affiliations:** 1 Department of Medicine, University of Ottawa, Ottawa, Ontario, Canada; 2 Faculty of Medicine, University of Ottawa, Ottawa, Ontario, Canada; 3 Department of Innovation and Medical Education, University of Ottawa, Ottawa, Ontario, Canada; University of Saskatchewan, CANADA

## Abstract

**Background:**

Illness presenteeism (IP) is the phenomenon where individuals continue to work despite illness. While it has been a prevalent and longstanding issue in medicine, the recent onset of the COVID-19 pandemic and the growing movement to improve physician wellness brings renewed interest in this topic. However, there have been no comprehensive reviews on the state of literature of this topic.

**Purpose:**

The main aim of this scoping review is to explore what is known about presenteeism in physicians, residents, and medical students in order to map and summarize the literature, identify research gaps and inform future research. More specifically: How has illness presenteeism been defined, problematized or perceived? What methods and approaches have been used to study the phenomenon? Has the literature changed since the pandemic?

**Method:**

Using the Arksey and O’Malley framework several databases will be searched by an experienced librarian. Through an iterative process, inclusion and exclusion criteria will be developed and a data extraction form refined. Data will be analyzed using quantitative and qualitative content analyses.

**Potential implications of results:**

By summarizing the literature on IP, this study will provide a better understanding of the IP phenomena to inform future research and potentially have implications for physician wellness and public health.

## Introduction

Illness Presenteeism (IP) is generally defined as the practice of going to work while experiencing poor physical or mental health [1] which can range in severity and acuity [2]. Although present in many professions, IP is more prevalent among healthcare workers (HCWs) compared with non-health-related occupations [3,4]. According to the 2010 European Working Conditions survey, 40% of the general population engaged in IP in the year prior to the study [5]. Similarly, a 2010 study of HCWs in New Zealand found that IP occurred in 48.7% of respondents. Remarkably, when compared to other HCWS including physiotherapists, nurses, and social workers, physicians were found to have the highest incidence of IP at 76.9%. Some studies report even higher numbers, with up to 80–90% of physicians reporting IP [6,7]. Thus—despite the potential adverse effects on patient care through the spread of contagious diseases, medical errors, worsened physician-patient communication, and decreased workplace productivity and efficiency—physicians appear to be the most frequent and notable “culprits” when it comes to IP [3,8,9]. Problematically, although it may also impair physicians’ and trainees’ personal health [9,10], IP has largely been overlooked in reforms or interventions aimed at addressing the well-being crisis in medicine.

The cultural milieu of medicine may provide some insight regarding higher rates of presenteeism among physicians specifically. Physicians sometimes identify as “anomalous patients”, viewing themselves as distinct from “ordinary patients” because of inherent power differentials in the patient-physician relationship [11]. Field et al. have recently shown that attending and resident physicians were reluctant to recognize the negative impact of working while impaired by fatigue despite evidence pointing to problems with patient care [12]. The competitive work environment seen in medicine may have a role to play in IP as well [13]. In a comparative study of IP among physicians in four European countries [14], a higher prevalence of IP was seen in Italy when compared to Sweden, Norway, and Iceland. Attendance and productivity pressures may account for differences. Using publication output as a surrogate measure, the higher prevalence of IP in Italy was associated with a more competitive work climate, with Italian physicians reporting more than twice as many published articles as physicians in other countries. Given these sociocultural factors within medicine, it is unsurprising that IP is also prevalent among physicians in training [15,16].

While IP is a longstanding practice in medicine, recent events have sparked renewed interest in this phenomenon. Physician health and well-being have accrued more attention in recent years, with a heightened focus to improve residency curricula and more research being conducted on burnout prevention and wellness [17,18]. In addition to this, the recent onset of the coronavirus disease 2019 (COVID-19) pandemic raised the stakes of transmitting a respiratory illness [19]. Indeed, during the height of the pandemic, policies were enacted that strictly forbade physicians from presenting to work sick to prevent infectious spread [20].

Existing research on IP has explored different aspects, including its prevalence [12], significant reasons and risk factors leading to presenteeism [2,3], and its associated consequences such as the potential compromise of patient safety due to impaired judgment, decreased functioning, job burnout, and loss of work productivity [9]. Three recent reviews focused on specific aspects of IP but precluded a fulsome understanding of this phenomena within the medical education context. The first is a narrative review [1] that summarized risk factors and economic and health consequences of IP in HCWs. Some contributing factors identified included the values entrenched within medical culture such as competitiveness, the idea that HCWs never fall ill, lack of medical resources, and strict sick leave policies [1,3,21]. However, this narrative review from 2019 provided a limited overview of the topic, restricted the search to a single database (PubMed), used limited search terms, and did not use an information specialist. The second systematic review [22] focused on specific questions on how to measure both the contributing factors to and outcomes of IP among hospital physicians and nurses. It provided a comprehensive tally of measurement instruments and workplace-related theoretical frameworks used in some studies they reviewed, it provided limited insight into the problem. Finally, a third review was not limited to physicians and studied IP with infectious illnesses in various occupations (this included HCWs of which some studies involving residents and medical students were included). While it provided insights on the self-reported motivations for infectious IP, the heterogeneity within the study limits our ability to translate these findings within a medical education context. Thus, although recent reviews on presenteeism do provide some insights, they were either of poor methodological quality [1] or were of narrow scope [3,22] and consequently did not provide an overview on the general state of the literature regarding IP among physicians. Moreover, these reviews primarily highlighted HCWs in general rather than focusing on physicians and physician trainees. They also do not account for pandemic-related shifts in IP perceptions and behaviorsIP in medicine demands attention, but thoughtful and robust research depends on first understanding the current state of knowledge. And while reviews on IP exist, their scope does not sufficiently capture a clear understanding of IP within the medicine and the medical education context in the current social context described above.

### Objective

Therefore, because a broad synthesis on IP in medicine is lacking, the purpose of this study is to explore what is known about presenteeism among physicians, residents, and medical students. A scoping review is an excellent tool to map the key concepts contained in a research domain—their breadth, limits, and features—and the primary sources and types of available evidence [23]. By summarizing this literature, we aim to identify gaps in the (potentially evolving) understanding on IP among physicians and physicians in training.

## Materials and methods

We will use the Arksey and O’Malley scoping review framework [24] with additional insights from Levac and colleagues [25]. We will follow the PRISMA-ScR checklist (S1 File) for reporting scoping reviews [26].

### Step 1: Identifying research question

The research team is starting with a broad research question: “What is known on illness presenteeism among physicians, residents, and medical students?” Through preliminary searches, several relevant articles were identified from the pandemic era [20,27,28]. After reviewing these articles, we developed the following specific questions:

- How has illness presenteeism been defined and problematized?
- What methods have been used to study the phenomenon of illness presenteeism among physicians and physicians in training?
- How have the perspectives on and implications of illness presenteeism changed since the pandemic?

### Steps 2 and 3: Identifying relevant studies and article selection

This scoping review will include all English-language published articles in healthcare and health professions education literature in any geographical region (context) directly addressing the topic of illness presenteeism (concept) among practicing independent physicians, residents, and/or medical students (participants). Moreover, we have not included any time constraints to allow us to capture the literature pre- and post-pandemic.

Several pilot searches will be completed by an information specialist. As described in the Joanna Briggs Institute (JBI) Manual for Evidence Synthesis [29] we will conduct a limited search in one database and analyze text words from the titles, abstracts, and index terms of relevant papers. Next, a second search will be conducted in all databases using all identified keywords. We will also review reference lists of all included studies to identify further relevant studies. To ensure rigour, the search strategy will be peer-reviewed by another librarian using the Peer Review of Electronic Search Strategies (PRESS) checklist [30]. Since we are interested in pre and the pandemic era, no date limits will be applied. A preliminary search strategy generated in conjunction with our information specialist (MD) is included in S2 File. Databases searched will include MEDLINE(R), Embase (OvidSP), APA PsycInfo (OvidSP), and Web of Science. We will also perform a hand-picked search of relevant grey literature related to IP. We will also include that were identified through reference searching of articles included in our search.

Article selection will proceed as follows. Using preliminary inclusion and exclusion criteria the entire team will review a random sample of 20 titles and abstracts to pilot the screening process. Refinements will be made in an iterative fashion, and we anticipate that it will require 2–3 team meetings. Thereafter, two team members will review the abstracts and titles arising from the search and apply the finalized inclusion/exclusion criteria with any conflicts being resolved by a third team member. The same process will be done for full-text articles. The following outlines our preliminary inclusion and exclusion criteria for screening titles and abstracts:

### Inclusion criteria

- English language
- Full-text original articles
- Original research related to working while physically or mentally ill, and may be labelled in many ways such as “illness presenteeism” or “sickness presenteeism”
- Include studies on physicians, resident physicians, and medical students
- May include any scholarly article types including perspective/commentary, editorials, reviews, or original research articles
- Any country
- We will also include grey literature related to major national North American physician organizations including the Association of Faculties of Medicine of Canada, the Royal College of Physicians and Surgeons of Canada, the Association of American Medical Colleges, and the Accreditation Council for Graduate Medical Education.

### Exclusion criteria

- Conference proceedings or published abstracts
- Full text articles not available

Full texts will be retrieved automatically from the Covidence software if available. The remaining full text articles will be searched and retrieved through our institution’s library, which also uses the Omni database—a resource that links several participating Ontario university libraries. Given the size of this database, articles that are unavailable within the database will be excluded from our scoping review. With respect to grey literature, we recognize that there may exist many global organizations that may have statements related to IP. To ensure that our search strategy remains feasible, we will limit our grey literature search to North American physician organizations.

All screening of titles/abstracts and full text reviews will be done using Covidence software. To date, the most recent search was conducted on May 29, 2023.

### Step 4: Charting (extracting) the data

#### Data extraction

We will develop a preliminary data extraction form based on our review of the literature. A draft can be seen in S3 File. The form will collect demographic information including publication year, journal, country of origin, and type of article. We will also extract the methods used in the article including whether the studies were quantitative, qualitative, or mixed methods and further specifying the subtype of study (e.g., cross-sectional, systematic review, etc.). In addition, we will seek information regarding physician level of training, specialty, practice setting (hospital vs ambulatory care), academic affiliation with university vs community setting), and illness type. Regarding IP, we will extract relevant information regarding how IP was defined, contributors to IP, consequences of IP (for patients, physicians and trainees, and the health care system—whether positive or negative), and any proposed solutions to IP presented by the articles being extracted. We will also identify any theoretical approaches used by various authors to frame their studies as part of the data extraction.

The data extraction form will be refined iteratively over several meetings. As outlined by Levac and colleagues [25], each revised version will be piloted on 6 articles reviewed by 2–4 members of the research team. This will be done until there has been consensus on a final data extraction form. Once the data extraction form is finalized, 30 articles will be independently coded by 2 team members and team meetings will ensure common understanding, and consistency. Thereafter one member of the team will complete the data extraction with a second independent member providing verification.

### Step 5: Collating and reporting findings

The articles included will undergo quantitative analyses which will involve descriptive numerical summary analysis of demographic data collected. Through qualitative thematic analysis, three members of the research team will independently review qualitative data to identify what Braun and Clarke would refer to as “manifest codes,” and thereafter develop preliminary themes that address our research question and objectives. Over several team meetings these themes will be explored and refined. Given the nature of our research questions, we plan to apply an inductive and semantic approach (i.e., analysis exploring meaning the surface, explicit, or manifest level) to describe and summarize our findings [31].

Depending on the richness of the data extracted, we may consider using existing theories that could be helpful in framing our findings. One example would be Expectancy Theory [32] which is a theory on the motivations behind work-related decisions made by individuals. This has previously been applied by Shweiki and colleagues [33] towards understanding resident physicians’ motivation during their training. Group members will review all available data and agree on a final summary of findings.

### Step 6: Consultations with stakeholders and potential knowledge users

While step 6 is outlined as an optional step in the Arksey and O’Malley framework, we will not pursue it during this review.

## Discussion

To our knowledge, this will be the first scoping review exploring the illness presenteeism amongst physicians and physician trainees. The identification and data synthesis will involve several databases including MEDLINE(R) ALL (OvidSP), Embase (OvidSP), APA PsycInfo (OvidSP), and Web of Science to reduce the risk of potential missed publications. This will be supplemented by relevant grey literature that will enrich our data further. To clarify, while we realize the scope of grey literature is limited, the organizations chosen were what the authors were most familiar with as it was not feasible to do a search of every international physician organization. The study will adhere to the steps outlined by Arksey and O’Malley and the PRISMA-SCR checklist to ensure rigour in our review. This study will be limited to English-language studies which may limit generalizability and bias results. This review may not find solutions to the phenomenon of IP but may better define the literature and the problem to contextualize future studies.

IP is a challenging issue in medicine and medical education as it deals with complex tensions and interactions between the health and wellbeing of physicians, their colleagues, and their patients. This phenomenon affects most physicians and is relevant to the current discourse of physician wellbeing and the COVID-19 pandemic. We anticipate that our findings will not only inform future research but will also raise awareness about—and challenge—sociocultural norms and behaviours like IP that may impair the well-being of physicians and trainees alike. Our scoping review may reveal structural biases or inequities that advantage and disadvantage certain individuals when navigating decision-making around whether to presenting to work when sick.

## Supporting information

S1 Checklist PRISMA-P 2015 checklist. (DOCX)

S1 File Appendix A: Prisma-ScR checklist. Preferred Reporting Items for Systematic Reviews and Meta-Analysis Extension for Scoping Reviews (PRISMA-ScR) Checklist.
(PDF)

S2 File Appendix B: Database search strategy. (DOCX)

S3 File Appendix C: Data extraction tool. (DOCX)
