# Supplementary material for: Illness presenteeism among physicians and trainees: Study protocol of a scoping review
Source: PLoS One. 2024 Feb 29;19(2):e0297447. doi: 10.1371/journal.pone.0297447 (PMC10903841; doi:10.1371/journal.pone.0297447)
Supplement: S2 File — (DOCX) [file pone.0297447.s003.docx]

**APPENDIX B: Search Strategy**

| **#** | **Query** |
| --- | --- |
| 1 | Presenteeism/ |
| 2 | presenteeism.ti,ab,kf. |
| 3 | ((work? Adj2 ill$4) or (work? Adj2 atten$4) or (work? Adj2 sick$4) or (sick$4 adj2 presen$4) or (sick$4 adj2 attend$4) or (presen$4 adj2 ill$4) or (unwell adj2 (doctor? Or physician?))).ti,ab,kf. |
| 4 | or/1-3 |
| 5 | exp medical staff/ or exp physicians/ |
| 6 | clinical clerkship/ or education, medical, graduate/ or “internship and residency”/ or education, medical/ or education, medical, undergraduate/ or teaching rounds/ |
| 7 | (doctor* or physician* or clinician* or specialist* or resident* or medical student* or surgeon* or hospitalist* or practitioner?).ti,ab,kf. |
| 8 | or/5-7 |
| 9 | 4 and 8 |
| 10 | limit 9 to English |
