# Supplementary material for: Illness presenteeism among physicians and trainees: Study protocol of a scoping review
Source: PLoS One. 2024 Feb 29;19(2):e0297447. doi: 10.1371/journal.pone.0297447 (PMC10903841; doi:10.1371/journal.pone.0297447)
Supplement: S3 File — (DOCX) [file pone.0297447.s004.docx]

**APPENDIX C: Data Extraction Tool**

Demographics:

- Author (Last Name)
- Publication Year
- Title
- Journal
- Country/Region
- Reviewer Initials
- Date of Review
- Article Type (Observational, Review, Commentary)
- Pre pandemic or during pandemic

Illness Presenteeism:

- How presenteeism was defined
  - Did the definition include poor performance
- What types of illness (e.g., physical and job related (such as overuse), physical not job related, mental health related)
- How was presenteeism measured?
- Research question/purpose of the paper
- Methods used
- Population Studied
  - Physician Level of Training (Attending, Resident, Medical Student)
  - Physician specialty
  - Form of Renumeration
  - Practice Setting (Hospital vs ambulatory care; academic vs community setting)
- Contributing Factors Studied (I.e., What exposures were associated with more or less presenteeism?)
- Outcomes Studied (e.g., effects on the individual, the individual’s colleagues, individual’s patients, individuals' workplace; economic implications)
- Study conclusions
